# The study on the impact of sex on the structure of gut microbiota of bamboo rats in China

**DOI:** 10.3389/fmicb.2023.1276620

**Published:** 2023-12-18

**Authors:** Yang Gan, Yan-jun Wu, Yuan-qiu Dong, Qian Li, Shu-guang Wu, Yuan-qing Jin, Tao-feng Lu

**Affiliations:** ^1^Guizhou University of Traditional Chinese Medicine, Guiyang, China; ^2^Kaili Hospital of Traditional Chinese Medicine, Kaili, Guizhou, China

**Keywords:** bamboo rat, 16S, gender, gut microbiome, cellulases producing bacteria

## Abstract

**Introduction:**

Bamboo rats are rodents that eat bamboo, and their robust capacity for bamboo digestion is directly correlated with their gut flora. Chinese bamboo rat (*Rhizomys sinensis*) is a common bamboo rat in Chinese central and southern regions. As a single-stomach mammal, bamboo rats are a famous specificity bamboo-eating animal and their intestinal microbial composition may also play a key role in the digestion of cellulose and lignin. So, the gut microbiota of bamboo rat may play an important role in the adaptation of bamboo rats for digesting lignocellulose-based diet.

**Methods:**

To study the microbiome differences of bamboo rats from different sexes, the microbial genomic DNA was extracted from each fecal sample and the V4 region of 16S rRNA genes was amplified and sequencing on an IlluminaHiSeq6000 platform. The operational taxonomic units (OTUs) were classified, the OTUs in different sexes was identified and compared at phylum and genus levels. For isolation and screening of cellulose degradation bacteria from bamboo rats, fresh feces from randomly selected bamboo rats were collected and used for the isolation and screening of cellulose degradation bacteria using Luria Bertani (LB) Agar medium containing Carboxymethyl cellulose. The cellulase activity, biochemical characterization and phylogenetic analysis of the purified bacteria strains were characterized.

**Results and discussion:**

A total of 3,833 OTUs were classified. The total microbial diversity detected in the female and male rats was 3,049 OTUs and 3,452 OTUs, respectively. The Shannon index revealed significant differences between the two groups (*p* < 0.05), though they were all captive and had the same feeding conditions. At the phylum level, *Firmicutes, Bacteroidota*, and *Proteobacteria* were prominent in the microbial community. At the genus level, the microbial community was dominated by *Lachnospiraceae, Lactobacillus, Bacteroides*, and *Prevotella*, but there was a significant difference between the two groups of bamboo rats; ~90 bacteria genus in the female group was significantly higher than the male group. Among them, *Bacteroides, Colidextribacter*, and *Oscillibacter* were significantly higher genera, and the genera of *Lachnoclostridium, Oscillibacter*, and *Papillibacter* had the highest FC value among the male and female bamboo rats. The KEGG function annotation and different pathways analysis revealed that membrane transport, carbohydrate metabolism, and amino acid metabolism were the most enriched metabolic pathways in the two groups, and multiple sugar transport system permease protein (K02025 and K02026), RNA polymerase sigma-70 factor (K03088), and ATP-binding cassette (K06147) were the three different KEGG pathways (*p* < 0.05). Two cellulose degradation bacteria strains—*Bacillus subtilis* and *Enterococcus faecalis*—were isolated and characterized from the feces of bamboo rats.

## 1 Introduction

A bamboo rat is a type of fossorial muroid rodent and gets its characteristic name from eating bamboo (Zhan et al., 2009). Bamboo rats belong to the subphyla Vertebrata, class Mammalia, order Rodentia, and family Rhizomyidae in animal taxonomy. The Rhizomyidae family has three main genera—Tachyoryctes, Cannomys, and Rhizomys, of which the Rhizomys genus has three species, namely the Chinese Bamboo Rat (*Rhizomys sinensis*), the Hoary Bamboo Rat (*Rhizomys pruinosus*), and the Large Bamboo Rat (*Rhizomys sumatrensis*) in China (Xu et al., 2016; Guo Y. T. et al., 2021). Bamboo rats are distributed in many provinces of China, such as Yunnan, Guizhou, Guangdong, and Guangxi, and the Chinese bamboo rat is common in the middle and southern regions of China. This species is a large solitary living rodent weighing approximately 1–4 kg, with a body length of 25–35 cm, a gray back and abdomen, small eyes and ears, and short limbs, living in dark and cool burrows underground in the wild. They inhabit bamboo thickets, pine forests, or evergreen broad-leaved forests at altitudes between 1,500 and 2,800 meters (Plestilova et al., 2016). The diet of Chinese bamboo rats includes shoots and roots of bamboo in addition to sugarcane or tapioca in cultivated lands (Wannaprasert, 2018).

Since 1990, the Chinese bamboo rat has been gradually domesticated and cultured in captivity (Ma et al., 2018). Besides, the bamboo rat breeding industry has gained widespread attention because of the deliciousness of their meat. However, due to changes in habitats and food sources, the transmission risk of zoonotic pathogens, such as *Cryptosporidium spp*. (Li et al., 2020b), *Penicilliosis marneffei* (Huang et al., 2015), *Giardia duodenalis* (Ma et al., 2018), *Enterocytozoon bieneusi* (Zhao et al., 2023), *Talaromyces marneffei* (Qu et al., 2023), and SARS-CoV-2 (Chen et al., 2022) has been reported in bamboo rats. Infections from these pathogens could seriously affect the health and economic significance of these animals (Guo Y. et al., 2021). After the COVID-19 outbreak, the captive breeding of bamboo rats was banned to avoid the risk of new and re-emerging infectious diseases. Currently, the Chinese bamboo rat is the most widely distributed species of bamboo rat, with a stable population and no survival crisis.

The animal gut is a complex digestive system. The host-associated microbial communities include various microorganisms (bacteria, archaea, fungi, and protozoa) and viruses (Hallen-Adams and Suhr, 2017). The microbial composition is influenced by host genetic factors (Kurilshikov et al., 2021), disease (Sanchez et al., 2015), diet, and gender (He et al., 2019). Bamboo rats mainly feed on bamboo, a low-nutrition food with approximately 70–80% of its composition being lignocellulosic components (Hu et al., 2017) that the single-stomach mammal cannot digest. Their ability to digest cellulose and lignin in bamboo largely depends on the composition of their gut microbiota (Xiao et al., 2022), which, consequently, may play an important role in their adaptation to digesting lignocellulose-based diets.

The phylogenetic composition of gut microbiota is influenced by diverse factors that include host gender. Sex biases in the gut microbiome drive host metabolism (Xie et al., 2017), the regulation of autoimmunity (Markle et al., 2013), and the response to various diseases (Krumsiek et al., 2015). For instance, in pigs, host gender significantly influences the phylogenetic composition of the gut bacterial community (Xiao et al., 2016). Currently, the bamboo rat still receives attention as a valuable wild animal resource and experimental animal model (Cao et al., 2020). However, research on the bamboo rat is relatively scarce, and further studies on its intestinal microbes are needed.

The bamboo rat is a famous specificity bamboo-eating animal, and cellulose degradation bacteria (CDB) may play a key role in its intestinal microbial composition for digesting a lignocellulose-based diet. However, the adaptation and mechanism of bamboo rats for digesting lignocellulose-based diet remain poorly understood. CDB has been isolated by many scholars from animal gastrointestinal tract and feces (Sari et al., 2017; Li et al., 2020a; Zhao et al., 2021), but there has been little research on bamboo rats. Therefore, the screening, isolation, and identification of CDBs from bamboo rats would be of great significance.

In this study, the fecal microbiome of Chinese bamboo rats was analyzed using 16S rRNA gene sequence technology, and the microbial community from different sexes was compared. In addition, the CDB in the feces of bamboo rats was also isolated and screened, and the characteristics of lignocellulose-degrading enzymes were studied. This study may provide a basis for further understanding of the effects of sex on intestinal microbes of bamboo rats, and the CDBs from bamboo rats may play a role in the bio-utilization of cellulose resources.

## 2 Materials and methods

### 2.1 Animals and specimen collection

Animal breeding and care and all experiments followed the “Regulations for the Administration of Affairs Concerning Experimental Animals of China” (CNAS-CL60). All protocols and studies involving animals were conducted following the guiding principles of the Animal Care and Use Committee of Guizhou University of Traditional Chinese Medicine (permit No. 2019007).

All bamboo rats were purchased from an artificial breeding farm (26.4268 N, 106.8527 E) in Guizhou province and were fed at the Institute of Laboratory Animals of Guizhou University of Traditional Chinese Medicine. The captive breeding of bamboo rats was legal before the COVID-19 outbreak, but it is now banned. A total of 42 bamboo rats were used in this study, and none of the animals were in a state of breeding, pregnancy, or lactation. Three bamboo rats of the same sex were raised in a breeding unit. Fourteen breeding units (six male units and eight female units) were used for this research. Three grains of fresh feces from each bamboo rat in the same breeding unit were collected by freehand stimulation method. The three grains from the same breeding unit were mixed as a 16S rRNA sequencing sample, and a total of 14 sequencing samples were prepared and stored at −80°C until use.

### 2.2 Extraction procedures and 16S rRNA sequencing

All 16S rRNA sequencing samples were transported on dry ice to the Beijing Novogene company (Beijing, China) and stored at −80°C. Microbial genomic DNA was extracted from each fecal sample (0.2 g) using the CTAB/SDS method. The V4 region of 16S rRNA genes was amplified using the specific primers 515F 5′-CCTAYGGGRBGCASCAG-3′ and 806R 5′-GGACTACNNGGGTATCTAAT-3′ with the barcode. After PCR product quantification, qualification, mixing, and purification, sequencing libraries were generated using TruSeq^®^ DNA PCR-Free Sample Preparation Kit (Illumina, USA) following the manufacturer's instructions. The library quality was assessed on the Qubit@ 2.0 Fluorometer (Thermo Scientific) and Agilent Bioanalyzer 2100 system. Finally, the library was sequenced on an IlluminaHiSeq6000 platform, and 250 bp paired-end reads were generated. All sequences were deposited in the Sequence Read Archive (SRA) under accession PRJNA980421.

### 2.3 Data analysis

Paired-end reads were merged using FLASH (V1.2.7, http://ccb.jhu.edu/software/FLASH/), a very fast and accurate analysis tool that is designed to merge paired-end reads when at least some of the reads overlap the read generated from the opposite end of the same DNA fragment. The splicing sequences were called raw tags. Sequences were analyzed using default parameters in the QIIME software package (Caporaso et al., 2010). Chimeric sequences were detected and removed using the UCHIME algorithm. Then, the effective tags were finally obtained. These sequences were grouped into operational taxonomic units (OTUs) using Uparse software (Uparse v7.0.1001, http://drive5.com/uparse/) with a minimum sequence identity of 97%. The most abundant sequences within each OTU were designated as the representative sequence, and then the annotation taxonomic information was obtained against the Silva Database (Quast et al., 2013) (http://www.arb-silva.de/) based on the Mothur algorithm. MUSCLE software (Version 3.8.31) was used to study the phylogenetic relationship of different OTUs.

The information on OTU abundance was normalized using a standard sequence number corresponding to the sample with the least sequences. The relative abundance of the phylum and genus levels between male and female Chinese bamboo rats was analyzed using the stack image and heatmap tools on the online OmicShare platform (http://www.omicshare.com). The OTUs number in male and female rats was calculated at phylum and genus levels, and the percentage of different terms was calculated and presented in the stack image. The data was normalized using the Z-score method, the mean and standard deviation (SD) were calculated, and the fold change value (FC, mean/SD) was presented in a heatmap. Subsequent alpha and beta diversity analyses were performed based on this output normalized data. Alpha diversity was applied to analyze the complexity of the diversity of the sample of four indices, including Simpson, ACE, Chao 1, and Shannon. We conducted statistical tests of ANOVA, and Tukey HSD was used to test the statistical significance of alpha diversity indices and to confirm the significance of the two groups (Zhang et al., 2022). For beta diversity, principal coordinates analysis (PCoA) based on unweighted and weighted UniFrac distances (Lozupone and Knight, 2015) was applied to compare the gut bacterial community between male and female bamboo rats.

To study the difference in the bacteria genus between the male and female bamboo rats, the FC and *p-*value were calculated using the normalization relative abundance data at the genus level using the edgeR package (Robinson et al., 2010) in R software (Version 4.1.2). The volcano map was plotted using log2 (FC) as the X-axis and -log10 (*p*-value) as the Y-axis. The difference in bacteria genus between the male and female bamboo rats was further analyzed at a significance threshold of *p* < 0.05 and log2 (FC) >1. The top 10 bacteria genera were plotted using the Omicshare tools, a free online platform for data analysis (https://www.omicshare.com/tools).

To study the main functions of the microbial community, the obtained clean tags were annotated using the PICRUSt program, and the Kyoto Encyclopedia of Genes and Genomes (KEGG) function was analyzed. Identified metabolites were annotated using the KEGG Compound database (http://www.kegg.jp/kegg/compound/), and annotated metabolites were then mapped to the KEGG Pathway database (http://www.kegg.jp/kegg/pathway.html). The relative abundance at the primary, secondary, tertiary, and KO levels of the KEGG pathway was analyzed. The difference pathways between the two groups were compared based on the normalization relative abundance data at a significance threshold of *p* < 0.05.

### 2.4 Isolation and screening of cellulose degradation bacteria

Eight healthy bamboo rats (four males and four females) from different breeding units were randomly selected for isolation and screening of cellulose degradation bacteria. Fresh feces of bamboo rats were collected by freehand stimulation method. About 1 g of fresh feces from each bamboo rat was diluted with sterilized phosphate buffer solution (PBS, pH 7.0), and serial dilutions from 10^−3^g/ml to 10^−6^g/ml were prepared using PBS. An aliquot of 50 μl of each dilution was incubated on Luria Bertani (LB) Agar medium containing 1% carboxymethyl cellulose (w/v), named LB-CMC Agar medium, under aerobic conditions at 37°C for 24 h.

Then, the Congo-red overlay method (Ariffin et al., 2008) was used to qualitatively screen the cellulose degradation bacteria. Specifically, the plates were flooded with about 10 ml Congo red stain (0.1%, w/v) for 30 min and then de-stained with 1N NaCl solution until the clear zones around the colonies were visualized. Based on visual inspection of colony morphologies such as shape, color, margin, and zone diameter, 2–3 colonies with similar morphology were picked and transferred to LB-CMC Agar medium. The selected bacterial colonies were further purified by streaking onto a new LB-CMC Agar medium for 4–5 generations. The purified bacteria strains were stored at −80°C with glycerol.

### 2.5 Preparation of crude enzyme solution and determination of the cellulose activity

The purified bacteria strains were activated and cultured in Luria-Bertani (LB) solid medium containing (g/L) NaCl (10.0), tryptone (10.0), yeast powder (5.0), and agar powder (10.0), under aerobic conditions at 37°C for 12 h. Then, the activated single colony was inoculated in Luria-Bertani (LB) liquid medium containing (g/L) NaCl (10.0), tryptone (10.0), and yeast powder (5.0) under aerobic conditions at 220 rpm and 37°C for 12 h. After that, they were cultured twice in LB liquid medium with 1.0% inoculum, and the optical density (OD) of the bacterial solution was adjusted to 1.0 at 600 nm (OD600 = 1.0) using LB liquid medium. The bacterial solution (0.5 ml) was inoculated in a 50 ml LB-CMC liquid medium containing 1% carboxymethyl cellulose (w/v) and was cultured at 220 rpm and 37°C for 24 h. The crude enzyme solution was obtained from the superstratum of the LB-CMC liquid medium and centrifuged at 3,000 g for 15 min.

The cellulose activity in the crude enzyme solution was detected by the 3,5-dinitrosalicylic acid (DNS) method (He et al., 2022). The degradation ability for endo-1,4-β-glucanase (CMCase) was detected using cellulose substances CMC. One unit (U) of enzyme activity was defined as the enzyme required for a substrate to produce 1 μg of glucose per minute.

### 2.6 Identification and phylogenetic study of the separated cellulose degradation bacteria

The genomic DNA from the overnight grown isolate was extracted by using the Rapid Bacterial Genomic DNA Isolation Kit from Sangon Biotech Co., Ltd. (Shanghai, China). The 16S rRNA gene was amplified by PCR from the cellulases producing bacteria genomic DNA using primers pairs, 7F (5′-CAGAGTTTGATCCTGGCT-3′) and 1540R (5′-AGGAGGTGTCCAGCCGCA-3′) (Zhang and Nan, 2012). Amplified products were checked for size and purity on 1% (w/v) agarose gel and were sequenced by Sangon Biotech Co., Ltd. (Shanghai, China). The obtained sequences were aligned using the Basic Local Alignment Search Tool (BLAST) program of NCBI for homology analysis against the Ribosomal database (http://rdp.cme.msu.edu/index.jsp). The nucleotide sequences of the isolate and closely related strains from GenBank were also used for alignment, and a Neighbor-Joining (NJ) phylogenetic tree was built using the MEGA 7.0 program (Kumar et al., 2016).

## 3 Results

### 3.1 Characterization of bacterial community from bamboo rats

**Figure 1:**
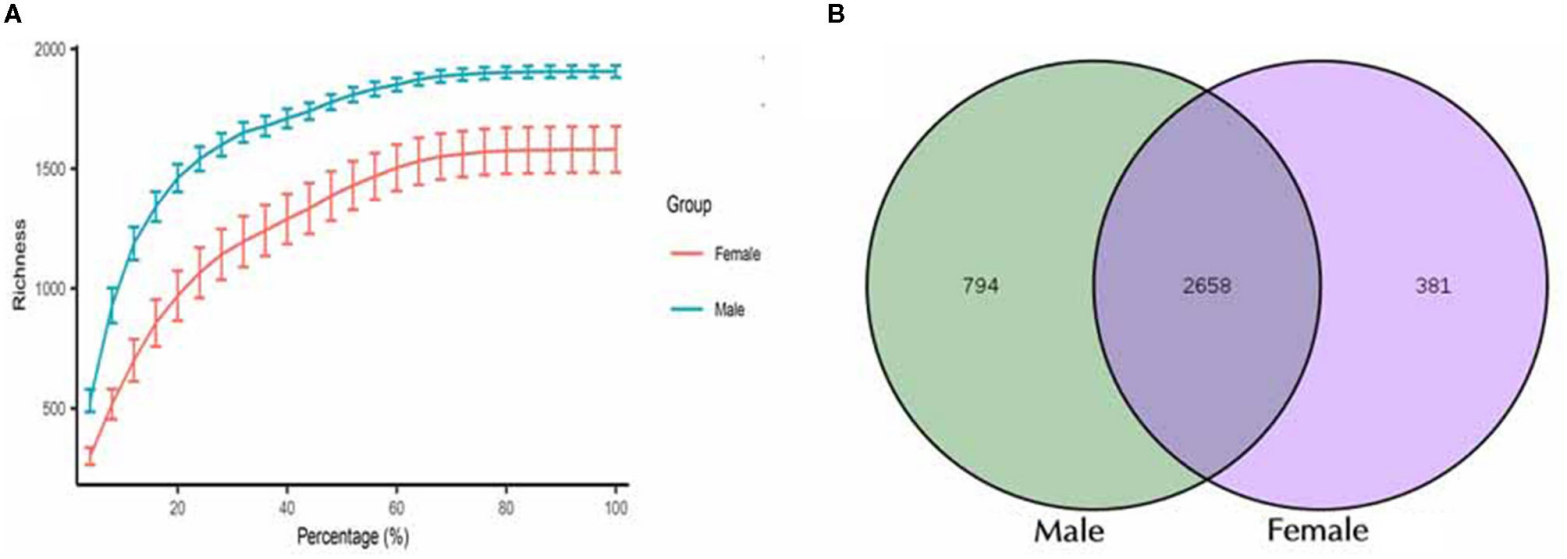
Characterization of sequencing data and the identified OTUs between male and female Chinese bamboo rats. **(A)** The rarefaction curves for the female and male groups. **(B)** Venn diagram of shared OTUs between male and female bamboo rats.

Sequencing analysis of 16S rRNA gene amplicons obtained 1,350,652 clean tags from all the tested samples (92,084–105,454 effective tags per sample). The length of the clean tags varied between 415 and 425 bp. Sequencing data quality was mainly distributed from 98.15 to 98.57% (Q20) to ensure normality of the subsequent advanced analysis. Rarefaction curves were calculated for the two groups (female and male) and are shown in Figure 1A. The two groups were sequenced sufficiently, and the curves reached complete saturation.

A total of 3,833 OTUs were classified, and these OTUs were used for all downstream analyses. The total microbial diversity detected in the female and male groups consisted of 3,049 and 3,452 OTUs, respectively. Analyzing all these OTUs, it was found that 2,658 OTUs were shared between the two groups (Figure 1B). The number of detected OTUs varied highly among samples (948–2,123 OTUs). The group with female rats harbored between 1,590 and 2,123 OTUs (median = 1,879 OTUs), while the one with the male rats had between 948 and 1,911 OTUs (median = 1,371 OTUs).

### 3.2 Microbial community analysis for the female and male bamboo rats

**Figure 2:**
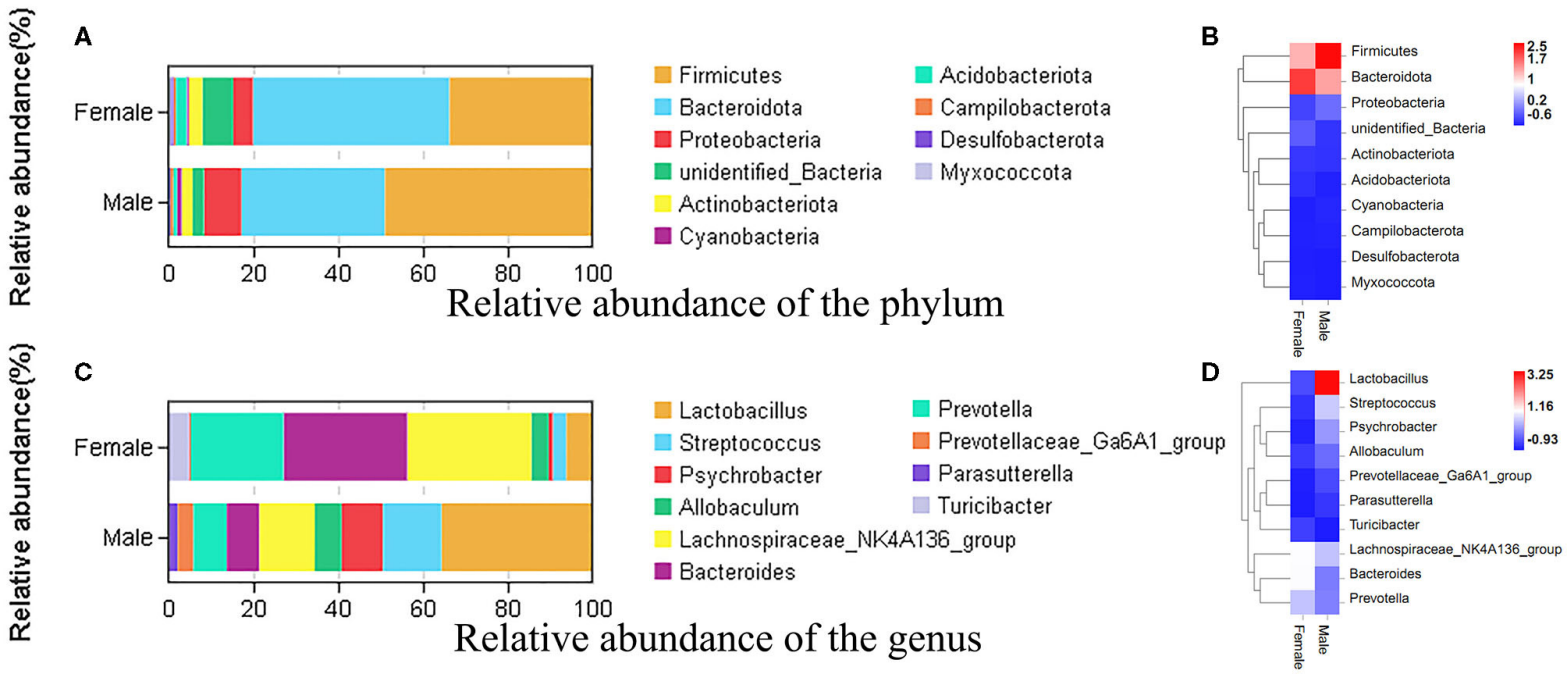
The species-relative abundance at the phylum and genus levels between male and female Chinese bamboo rats. **(A)** The stacked image at the phylum level. **(B)** The heat map at the phylum level. **(C)** The stacked image at the genus level. **(D)** The heat map at the genus level. The OTUs number in different sexes was calculated at phylum and genus levels, and the percentage of different terms was calculated and presented in the stack image. The data was normalized using the z-score method, and the fold change value (FC, mean/SD) was calculated and presented in a heatmap.

To study the microbial community diversity between the two sexes of bamboo rats, the relative abundance of the microbial community in the male and female groups was analyzed at phylum and genus levels. At the phylum level (Figure 2A), *Firmicutes* (33.8% for male and 49% for female), *Bacteroidota* (46.3% for male and 33.8% for female), *Proteobacteria* (4.7% for male and 8.8% for female), and *Actinobacteriota* (3.2% for male and 2.5% for female) were the main enrichment of the microbial community, and the relative abundance of other bacteria was lower than 1%. The heat map of the species-relative abundance at the phylum level (Figure 2B) also showed that *Firmicutes, Bacteroidota, and Proteobacteria* were the main enrichment of the microbial community.

At the genus level (Figure 2C), the microbial community was dominated by the *Lachnospiraceae* NK4A136 group (21.2%), *Lactobacillus* (20.9%), *Bacteroides* (18.4%), and *Prevotella* (14.8%) in the feces of bamboo rats. Specifically, the dominant microbial genus in female bamboo rats was the *Lachnospiraceae* NK4A136 group (29.3%), *Bacteroides* (29.1%), *Prevotella* (21.8%), and *Lactobacillus* (6.2%); the dominant microbial genus in the male bamboo rats was *Lactobacillus* (35.6%), *Streptococcus* (13.7%), the *Lachnospiraceae* NK4A136 group (13.1%), and *Psychrobacter* (9.8%). The heat map of the species-relative abundance at the genus level (Figure 2D) also showed obvious differences between male and female Chinese bamboo rats.

### 3.3 Different bacteria genera between male and female bamboo rats

**Figure 3:**
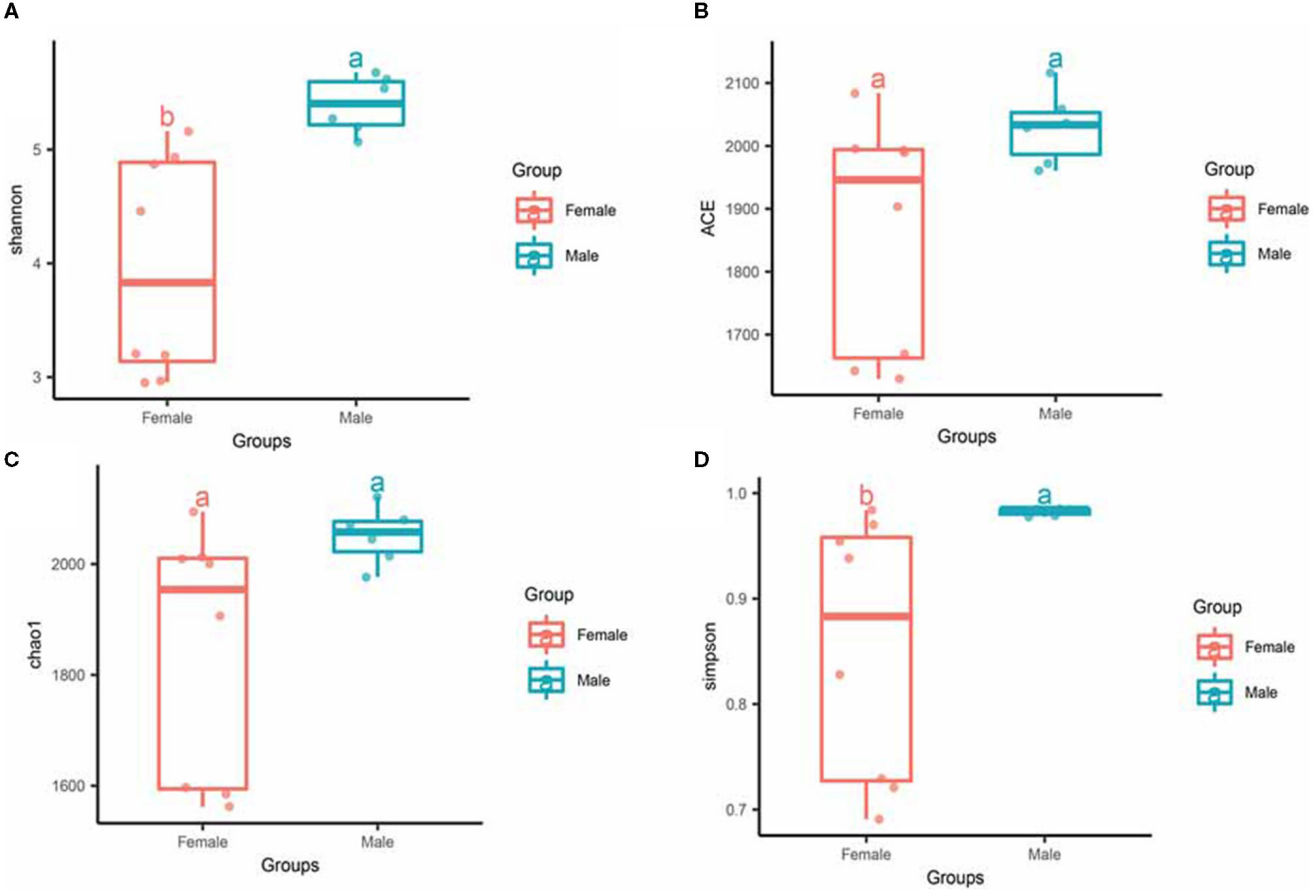
Alpha diversity of gut microbiota in male and female rats. The evenness of the bacteria community was evaluated by the Shannon **(A)**, ACE **(B)**, Chao1 **(C)**, and Simpson **(D)** index. Box plots show high, low, and median values, with each box's lower and upper edges denoting the first and third quartiles. The x-axis represents the information on samples. Statistical significance between different groups was indicated by a different letter (a or b); the same letters represent insignificant differences (*p* > 0.05), and different letters represent significant differences (*p* < 0.05).

To investigate the differences in microbial diversity between male and female bamboo rats, the normalized OTUs data of the two groups were used, and the values of the alpha diversity index (Simpson, ACE, Chao 1, and Shannon) were calculated (Figure 3). The Shannon index had a significant difference between the two groups (*p* < 0.05), and the other diversity index (ACE, Chao 1, Shannon and Simpson) did not differ significantly between the two groups (*p* > 0.05).

**Figure 4:**
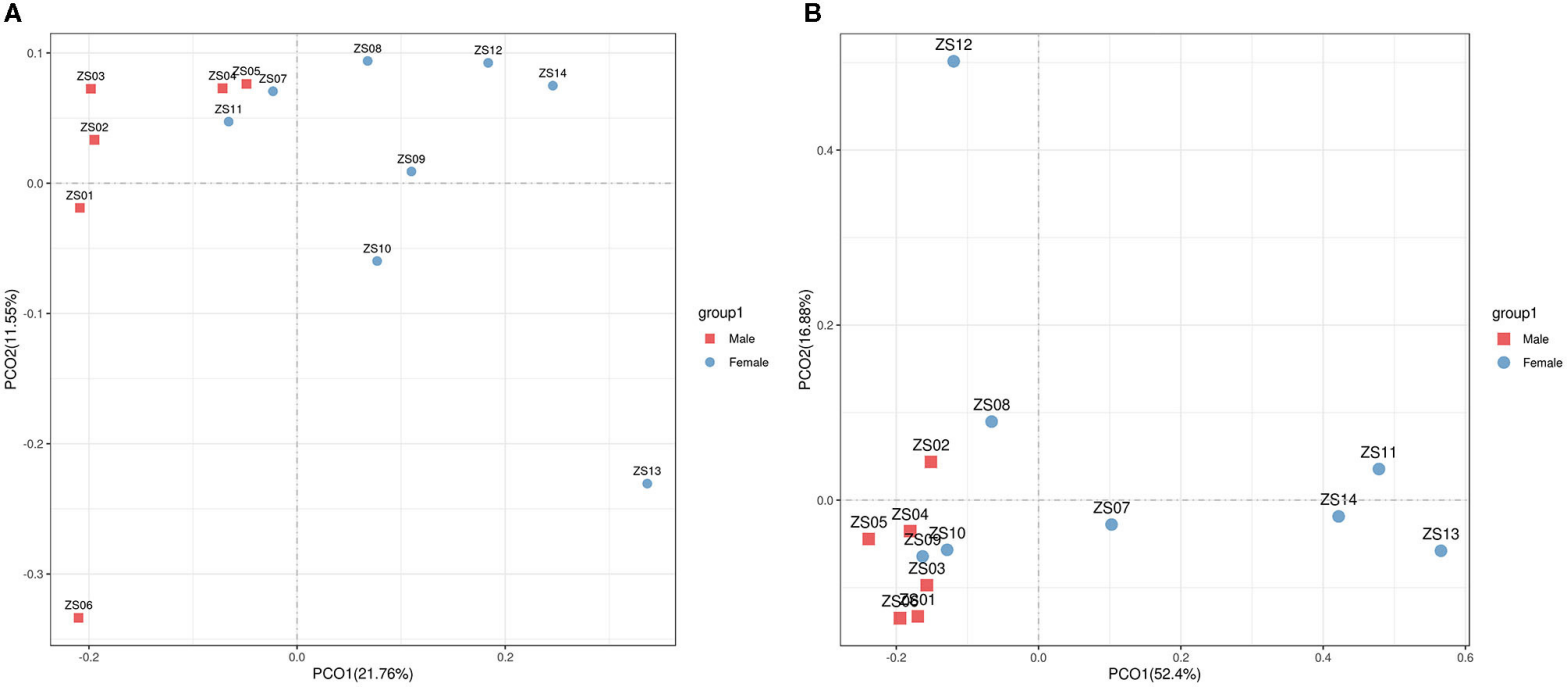
Beta diversity of gut microbiota in bamboo rats based on unweighted and weighted UniFrac distances. PCoA shows beta diversity based on unweighted **(A)** and weighted **(B)** UniFrac distances at the OTU level. The variation explained by the plotted principal coordinates is indicated in the axis label.

For beta diversity, PCoA based on unweighted and weighted UniFrac distances were applied to compare the gut bacterial community between the male and female groups of bamboo rats. We found that the samples from the male and female groups were individually clustered based on unweighted (Figure 4A) and weighted (Figure 4B) UniFrac distances, which indicated that the species-abundance distribution of the gut microbiota of the two groups was different from each other, even though they are all captive and under the same feeding conditions.

**Figure 5:**
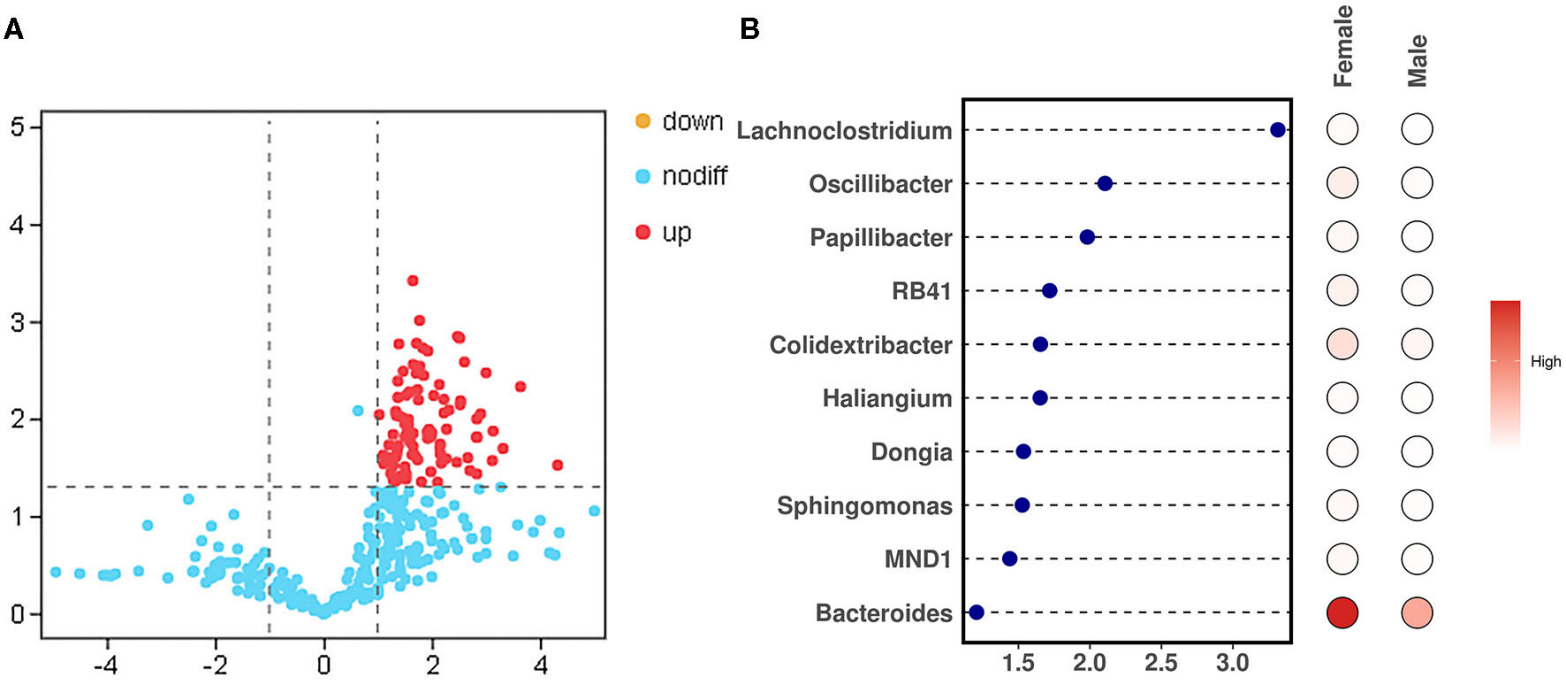
Different bacteria genera between the male and female bamboo rats. **(A)** The volcano map was plotted using the normalization relative abundance data at a significance threshold of *p* < 0.05 and log_2_ (FC) >1. **(B)** The point-bar heatmap was plotted using the top 10 bacteria genera between the male and female bamboo rats.

To study the difference in the bacteria genus between the male and female bamboo rats, the volcano map (Figure 5A) was mapped using log_2_ (FC) as the x-axis and –log_10_ (*p-*value) as the y-axis. The result indicated that 90 bacteria genera in female bamboo rats was significantly higher than that in male rats with *p* < 0.05 and log_2_ (FC) >1 using the normalization relative abundance data at the genus level. The top 10 bacteria genus is listed in Table 1. The result showed that the bacteria genus from the female group, such as *Bacteroides, Colidextribacter*, and *Oscillibacter*, was significantly higher than that of the male group at a significance threshold of *p* < 0.05 and log_2_ (FC) >1, and the bacteria genus, such as *Lachnoclostridium, Oscillibacter*, and *Papillibacter*, had the biggest FC value between the male and female bamboo rats (Figure 5B).

**Table 1:** Top 10 different bacteria genera between male and female bamboo rats.

**Bacteria genus**	**Female (%)**	**Male (%)**	**Log_2_ (FC)**	***p*-value**
*Bacteroides*	9.793	4.241	1.210	0.019
*Colidextribacter*	1.561	0.496	1.654	0.003
*Oscillibacter*	0.801	0.186	2.106	0.045
*RB41*	0.689	0.209	1.719	0.002
*Papillibacter*	0.373	0.095	1.982	0.015
*Sphingomonas*	0.357	0.124	1.527	0.006
*MND1*	0.341	0.126	1.440	0.010
*Lachnoclostridium*	0.277	0.028	3.315	0.020
*Dongia*	0.218	0.075	1.536	0.014
*Haliangium*	0.211	0.067	1.652	0.014

### 3.4 KEGG function annotation and analysis

**Figure 6:**
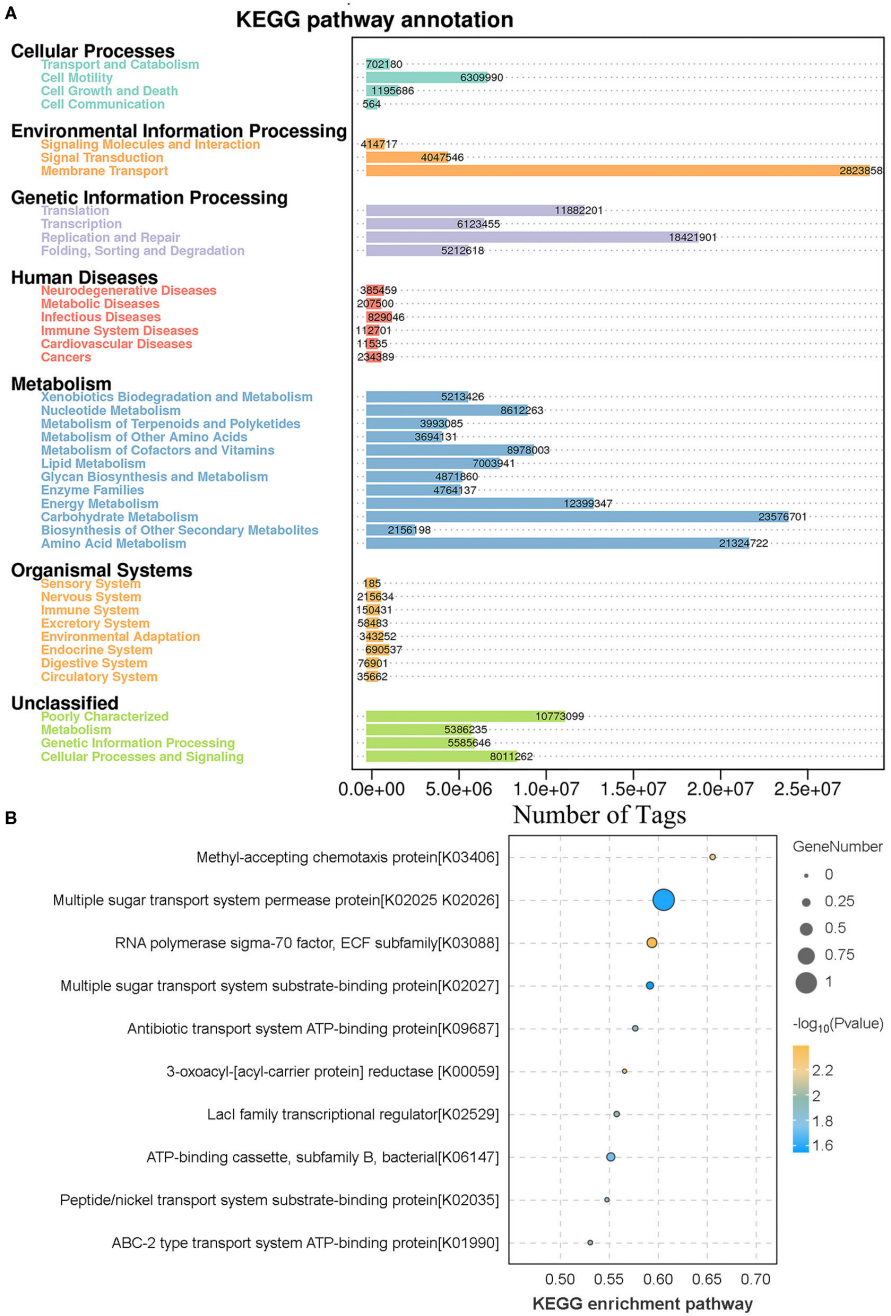
KEGG function annotation and analysis. **(A)** The clean tags enrichment in the KEGG annotation pathway. The clean tags were mapped to the KEGG Pathway database, and the number of clean tags was labeled. **(B)** The differences in KEGG enrichment pathway between male and female bamboo rats. The clean tags from male and female bamboo rats were normalized and analyzed, and the difference pathways between the two groups were compared at a significance threshold of *p* < 0.05. The top 10 different KEGG pathways are shown.

The results of KEGG analysis showed that 7 primary, 41 secondary, 299 tertiary, and 6,039 KO level pathways were identified. Among them, membrane transport (28,238,583 tags), carbohydrate metabolism (23,576,701 tags), and amino acid metabolism (21,324,722) were the most enriched metabolic pathways, and the results are shown in Figure 6A. To study the different KEGG pathways between female and male bamboo rats, the enriched KEGG pathways from the two groups were analyzed, and the top 10 different KEGG pathways at a significance threshold of *p* < 0.05 are shown in Figure 6B. The results showed that the top 3 different KEGG pathways (*p* < 0.05) were multiple sugar transport system permease proteins (K02025 and K02026, *p* = 0.026), RNA polymerase sigma-70 factor (K03088, *P* = 0.004), and ATP-binding cassette (K06147, *p* = 0.019).

### 3.5 Isolation and screening of CDB

**Figure 7:**
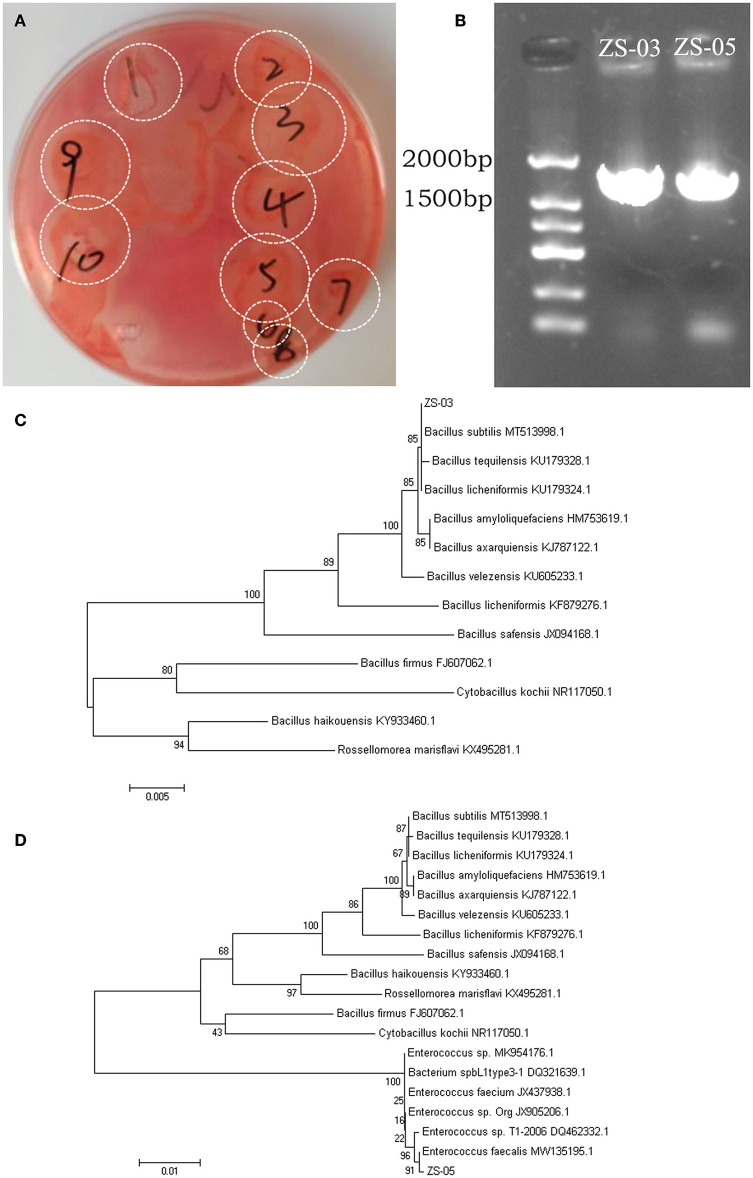
Screening and phylogenetic analysis for the isolated CDBs. **(A)** Cleaning zone of cellulolytic bacteria on CMC-Na selective medium after Congo red staining. **(B)** Electrophoresis of 16S rRNA gene PCR products for the two isolates—ZS-03 and ZS-05. **(C)** The NJ phylogenetic tree based on the data of the ZS-03 strain and the other bacteria strains, including *Bacillus subtilis* (MT513998.1), *Bacillus tequilensis* (KU179328.1), *Bacillus licheniformis* (KU179324.1), *Bacillus amyloliquefaciens* (HM753619.1), *Bacillus axarquiensis* (KJ787122.1), *Bacillus velezensis* (KU605233.1), *Bacillus licheniformis* (KF879276.1), *Bacillus safensis* (JX94168.1), *Bacillus firmus* (FJ607062.1), *Cytobacillus kochii* (NR117050.1), *Bacillus haikouensis* (KY933460.1), and *Rossellomorea marisflavi* (KX495281.1). **(D)** The NJ phylogenetic tree based on the data of the ZS-05 strain and the other bacteria strains, including *Bacillus subtilis* (MT513998.1), *Bacillus tequilensis* (KU179328.1), *Bacillus licheniformis* (KU179324.1), *Bacillus amyloliquefaciens* (HM753619.1), *Bacillus axarquiensis* (KJ787122.1), *Bacillus velezensis* (KU605233.1), *Bacillus licheniformis* (KF879276.1), *Bacillus safensis* (JX094168.1), *Bacillus haikouensis* (KY933460.1), *Rossellomorea marisflavi* (kX495281.1), *Bacillus firmus* (FJ607062.1), *Cytobacillus kochii* (NR117050.1), *Enterococcus* sp. (MK954176.1), *Bacterium* spbL1type3-1 (DQ321639.1), *Enterococcus faecium* (JX437938.1), *Enterococcus* sp.Org (JX905206.1), *Enterococcus* sp.T1-2006 (DQ462332.1), and *Enterococcus faecalis* (MW135195.1).

Bamboo rats are a famous specificity bamboo-eating animal, and their intestinal microbial composition may also play a key role in the digestion of cellulose and lignin. So, isolating and identifying cellulolytic bacteria from bamboo rats is very interesting and meaningful. After isolation and screening of CDB on LB-CMC Agar medium plates at 37°C for 24 h, cellulose-solvent zones around colonies were formed. In the study, many bacterial isolates from the Chinese bamboo rats were initially identified to produce fiber degrading enzymes on CMC Agar plates (Figure 7A). Out of these isolates, two isolates encoded as ZS-03 and ZS-05 showed a maximum clearance zone (25.7 mm and 24.6 mm, respectively) when stained with Congo red. Therefore, the two isolates were selected for further study. The cellulase activity and biochemical characterization of ZS-03 and ZS-05 are shown in Table 2.

**Table 2:** Cellulase activity and biochemical characteristics of ZS-03 and ZS-05.

**Characteristic features**	**Results**	
	**ZS-03**	**ZS-05**
Cellulase activity (U/mL)	29.87	28.23
Gram staining	+	+
Glucose utilization	+	+
Maltose utilization	+	+
Sorbitol fermentation	-	+
Mannitol fermentation	-	+
Salicin fermentation	+	-
Melampyrit fermentation	-	+
Malonate utilization	-	-
Citrate utilization	-	-
Methyl red	-	+
Voges-Proskauer	-	-
Indole	-	+
Urease	+	-
ONPG	+	+

### 3.6 Phylogenetic analysis

The agarose gel electrophoresis result of the PCR amplification product (using 7F and 1540R primers) of the strain ZS-03 and ZS-05 are shown in Figure 7B. The DNA fragments amplified by PCR were all single bands and about 1,500 bp. The PCR products of the two isolates, ZS-03 and ZS-05, were sequenced to obtain the length of 1,444 bp (GenBank ID: SUB13733541) and 1,457 bp (GenBank ID: SUB13733559) gene sequences, which was consistent with the electrophoresis result.

The homology between the ZS-03 strain and *Bacillus subtilis* (MT513998.1) was 99%, suggesting the ZS-03 strain was a close relative of *Bacillus subtilis* (Figure 7C). The homology between the ZS-05 strain and *Enterococcus faecalis* (MW135195.1) was 99%, suggesting the ZS-05 strain was a close relative of *Enterococcus faecalis* (Figure 7D).

## 4 Discussion

Gut microbes are closely related to the host's nutrient absorption and metabolism and can help the host perform specific physiological and biochemical functions (Zhang et al., 2023). Fecal samples are a non-invasive and sustainable method for observing animal gut microbiota. Although fecal samples cannot reflect the current dynamic changes of bacteria in the animal gut, they can still reflect the composition of the entire gut microbiota (He et al., 2019), which can help us understand the structure and differences of the gut microbiota of animals. The bamboo rat is a famous specificity bamboo-eating animal, and the gut microbiota of bamboo rats may play an important role in their adaptation to digesting lignocellulose-based diet.

In this study, 3,833 OTUs were classified by analyzing 16s rRNA amplicons from all fecal samples. The microbial community was dominated by *Lachnospiraceae, Lactobacillus, Bacteroides*, and *Prevotella* in the gut microbiotas of bamboo rats. Among them, *Lachnospiraceae, Lactobacillus*, and *Bacteroides* were the main microbial genus involved in lignocellulose utilization in bamboo rats (Xiao et al., 2022). *Prevotella* is well known as a dietary fiber fermenter, and the *Prevotella*-dominated microbiota are associated with complex carbohydrate consumption (Chen et al., 2017). So, in the gut of a bamboo rat, these associated bacterial members formed a strong inner-connected microbial community with richness in metabolic and fermentative functions. These findings indicate that the gut microbiotas of bamboo rats have a unique function to adapt to the utilization of complex lignocelluloses.

Host sex significantly influences the phylogenetic composition of the gut microbiota and drives host metabolism, the regulation of autoimmunity, and the response to various diseases. As a new potential resource for experimental animals, it is necessary to study the impact of sex on the structure of the gut microbiota of bamboo rats. In this study, the identified 3,833 OTUs were used to determine how sex variation affects the gut microbiota of bamboo rats. A similar study in pigs declared that the number of observable OTUs of gut microbiotas was insignificant between the two sexes. The α-diversity of the gut microbiota was significantly lower in females (He et al., 2019) even though they were all held under the same feeding conditions. At the taxonomic level and consistent with previous reports in bamboo rats (Xiao et al., 2022), the phyla *Firmicutes* (33.8% for males and 49% for females), *Bacteroidota* (46.3% for males and 33.8% for females), and *Proteobacteria* (4.7% for males and 8.8% for females) were the main enrichment of the microbial community of bamboo rats. These have been reported to substantially contribute to the taxonomic and metabolic variations in the gut microbiome of humans (Human Microbiome Project, 2012), ruminants (Xie et al., 2021), and giant pandas (Guo et al., 2020).

A previous study has indicated profound interactions between host sex and the gut microbiome (Yurkovetskiy et al., 2013) and the influence of androgens on gut microbial composition (Markle et al., 2013) in mice because of the bi-directional interaction between gut microbiota and sex hormones. For instance, *Clostridium scindens* can convert glucocorticoids to androgens (Ridlon et al., 2013), and the sex differences can be obscured by host genetics and environmental factors (Org et al., 2016). Many sex-biased bacteria have been reported in mice and humans. For example, *Lactobacillus, Veillonellaceae, Enterobacteriaceae, Roseburia, Eubacterium, Sutterella*, and *Coprococcus* represent sex-biased bacteria in mice (Yurkovetskiy et al., 2013; Org et al., 2016), while *Eubacterium, Blautia*, and *Treponema* represent sex-biased bacteria in humans (Schnorr et al., 2014). Likewise, *Treponema* and *Bacteroides* were over-represented in gilts (He et al., 2019), and it was found that Estrogen inhibited the overgrowth of *Escherichia coli* in rat intestine (Yang et al., 2018).

In this study, we found that *Lachnospiraceae, Lactobacillus, Bacteroides*, and *Prevotella* were the dominant microbial genus in the gut microbiotas of bamboo rats. *Lactobacillus* and *Bacteroides* have been previously mentioned as sex-biased microbial genera in mice (Yurkovetskiy et al., 2013) and pigs (He et al., 2019). Nevertheless, *Bacteroides, Colidextribacter*, and *Oscillibacter* were the significantly higher microbial genera based on the different bacteria genus analyses between the male and female bamboo rats. The bacterium from the *Colidextribacter* genus can produce inosine, which has a protective effect on LPS-induced acute liver injury and inflammation in mice (Guo W. et al., 2021). The bacterium from the *Oscillospira* genus involved in gluconate utilization is widely present in the digestive tract of herbivores and is involved in the butyrate kinase-mediated pathway (Chen et al., 2020). These findings indicate that *Bacteroides, Colidextribacter*, and *Oscillibacter* may represent sex-biased bacteria in bamboo rats, and the sex-biased microbial genus of bamboo rats is different from humans and other animals.

Bamboo rats are well-known for their unique ability to digest cellulose and lignin, which is attributed to their intestinal microbial composition. However, the adaptation and mechanism of bamboo rats for digesting lignocellulose-based diet remain poorly understood. The isolation and identification of CDBs from bamboo rats are pertinent to investigating the role CDBs may play in the bio-utilization of cellulose resources. Some CDBs have been isolated by many scholars from animal gastrointestinal tract and feces, such as *Bacillus velezensis* from Min pig (Li et al., 2020a), *Enterobacteriaceae* from bovine rumen fluid (Sari et al., 2017), and *Enterococcus faecalis* and *Enterococcus faecium* from Tibetan yak rumen (Zhao et al., 2021), but there are no related reports on the Chinese bamboo rat. In this study, two strains (ZS-03 and ZS-05) of cellulose-degrading bacteria from bamboo rats were isolated and characterized. The ZS-03 strain was classified as *Bacillus subtilis*, and the ZS-05 strain was classified as *Enterococcus faecalis*. The results of this experiment proved that it was feasible to isolate CDBs from the gastrointestinal tract system of bamboo rats.

## Data availability statement

The datasets presented in this study can be found in online repositories. The names of the repository/repositories and accession number(s) can be found in the article/supplementary material.

## Ethics statement

The study was approved by the Guizhou University of Traditional Chinese Medicine and performed following animal ethics guidelines and approved protocols. The animal ethics approval number is Guizhou SYXK-2019-007. The studies were conducted in accordance with the local legislation and institutional requirements. Written informed consent was obtained from the owners for the participation of their animals in this study.

## Author contributions

YG: Data curation, Visualization, Writing—original draft, Project administration. Y-jW: Data curation, Formal analysis, Software, Writing—original draft. Y-qD: Methodology, Project administration, Visualization, Writing—original draft. QL: Investigation, Methodology, Project administration, Writing—original draft. S-gW: Resources, Supervision, Validation, Writing—review & editing. Y-qJ: Methodology, Project administration, Resources, Writing—review & editing. T-fL: Funding acquisition, Methodology, Visualization, Writing—review & editing, Formal analysis, Writing—original draft.

## References

[B1] Ariffin H. Hassan M. A. Shah U. K. Abdullah N. Ghazali F. M. Shirai Y. (2008). Production of bacterial endoglucanase from pretreated oil palm empty fruit bunch by bacillus pumilus EB3. J. Biosci. Bioeng. 106, 231–236. 10.1263/jbb.106.231 18929997

[B2] Cao W. Pu P. Wang J. Niu Z. Zhang T. He J.. (2020). Suppressed LPS-mediated TLR4 signaling in the plateau zokor (*Eospalax baileyi*) compared to the bamboo rat (*Rhizomys pruinosus*) and rat (*Rattus norvegicus*). J. Exp. Zool. A Ecol. Integr. Physiol. 333, 240–251. 10.1002/jez.2346 31994847

[B3] Caporaso J. G. Kuczynski J. Stombaugh J. Bittinger K. Bushman F. D. Costello E. K.. (2010). QIIME allows analysis of high-throughput community sequencing data. Nat. Methods 7, 335–336. 10.1038/nmeth.f.303 20383131 PMC3156573

[B4] Chen P. Wang J. Xu X. Li Y. Zhu Y. Li X.. (2022). Molecular dynamic simulation analysis of SARS-CoV-2 spike mutations and evaluation of ACE2 from pets and wild animals for infection risk. Comput. Biol. Chem. 96, 107613. 10.1016/j.compbiolchem.2021.107613 34896769 PMC8634692

[B5] Chen T. Long W. Zhang C. Liu S. Zhao L. Hamaker B. R. (2017). Fiber-utilizing capacity varies in Prevotella- versus Bacteroides-dominated gut microbiota. Sci. Rep. 7, 2594. 10.1038/s41598-017-02995-4 28572676 PMC5453967

[B6] Chen Y. R. Zheng H. M. Zhang G. X. Chen F. L. Chen L. D. Yang Z. C. (2020). High Oscillospira abundance indicates constipation and low BMI in the Guangdong Gut Microbiome Project. Sci. Rep. 10, 9364. 10.1038/s41598-020-66369-z 32518316 PMC7283226

[B7] Guo W. Chen Y. Wang C. Ning R. Zeng B. Tang J.. (2020). The carnivorous digestive system and bamboo diet of giant pandas may shape their low gut bacterial diversity. Conserv. Physiol. 8, coz104. 10.1093/conphys/coz104 32190328 PMC7066643

[B8] Guo W. Xiang Q. Mao B. Tang X. Cui S. Li X.. (2021). Protective effects of microbiome-derived inosine on lipopolysaccharide-induced acute liver damage and inflammation in mice via mediating the TLR4/NF-kappaB pathway. J. Agric. Food Chem. 69, 7619–7628. 10.1021/acs.jafc.1c01781 34156842

[B9] Guo Y. Li N. Feng Y. Xiao L. (2021). Zoonotic parasites in farmed exotic animals in China: Implications to public health. Int. J. Parasitol. Parasites Wildl. 14, 241–247. 10.1016/j.ijppaw.2021.02.016 33898224 PMC8056123

[B10] Guo Y. T. Zhang J. Xu D. M. Tang L. Z. Liu Z. (2021). Phylogenomic relationships and molecular convergences to subterranean life in rodent family Spalacidae. Zool. Res. 42, 671–674. 10.24272/j.issn.2095-8137.2021.240 34490760 PMC8455469

[B11] Hallen-Adams H. E. Suhr M. J. (2017). Fungi in the healthy human gastrointestinal tract. Virulence 8, 352–358. 10.1080/21505594.2016.1247140 27736307 PMC5411236

[B12] He M. Gao J. Wu J. Zhou Y. Fu H. Ke S.. (2019). Host gender and androgen levels regulate gut bacterial taxa in pigs leading to sex-biased serum metabolite profiles. Front. Microbiol. 10, 1359. 10.3389/fmicb.2019.01359 31275280 PMC6591444

[B13] He Z. Ding B. Ali Q. Liu H. Zhao Y. Wang X.. (2022). Screening and isolation of cold-adapted cellulose degrading bacterium: A candidate for straw degradation and De novo genome sequencing analysis. Front. Microbiol. 13, 1098723. 10.3389/fmicb.2022.1098723 36713214 PMC9880256

[B14] Hu Y. Wu Q. Ma S. Ma T. Shan L. Wang X.. (2017). Comparative genomics reveals convergent evolution between the bamboo-eating giant and red pandas. Proc. Natl. Acad. Sci. U S A 114, 1081–1086. 10.1073/pnas.1613870114 28096377 PMC5293045

[B15] Huang X. He G. Lu S. Liang Y. Xi L. (2015). Role of *Rhizomys pruinosus* as a natural animal host of *Penicillium marneffei* in Guangdong, China. Microb. Biotechnol. 8, 659–664. 10.1111/1751-7915.12275 25824250 PMC4476820

[B16] Human Microbiome Project C. (2012). Structure, function and diversity of the healthy human microbiome. Nature 486, 207–214. 10.1038/nature11234 22699609 PMC3564958

[B17] Krumsiek J. Mittelstrass K. Do K. T. Stuckler F. Ried J. Adamski J.. (2015). Gender-specific pathway differences in the human serum metabolome. Metabolomics 11, 1815–1833. 10.1007/s11306-015-0829-0 26491425 PMC4605991

[B18] Kumar S. Stecher G. Tamura K. (2016). MEGA7: molecular evolutionary genetics analysis version 7.0 for bigger datasets. Mol. Biol. Evol. 33, 1870–1874. 10.1093/molbev/msw054 27004904 PMC8210823

[B19] Kurilshikov A. Medina-Gomez C. Bacigalupe R. Radjabzadeh D. Wang J. Demirkan A.. (2021). Large-scale association analyses identify host factors influencing human gut microbiome composition. Nat. Genet. 53, 156–165. 10.1038/s41588-020-00763-1 33462485 PMC8515199

[B20] Li F. Xie Y. Gao X. Shan M. Sun C. Niu Y. D.. (2020a). Screening of cellulose degradation bacteria from Min Pigs and optimization of its cellulase production. Electron. J. Biotechnol. 48, 29–35. 10.1016/j.ejbt.2020.09.001

[B21] Li F. Zhao W. Zhang C. Guo Y. Li N. Xiao L.. (2020b). Cryptosporidium species and *C. parvum* subtypes in farmed bamboo rats. Pathogens 9, 1018. 10.3390/pathogens9121018 33276616 PMC7761605

[B22] Lozupone C. A. Knight R. (2015). The UniFrac significance test is sensitive to tree topology. BMC Bioinform. 16, 211. 10.1186/s12859-015-0640-y 26150095 PMC4492014

[B23] Ma X. Wang Y. Zhang H. J. Wu H. X. Zhao G. H. (2018). First report of Giardia duodenalis infection in bamboo rats. Parasit. Vectors 11, 520. 10.1186/s13071-018-3111-2 30236164 PMC6149208

[B24] Markle J. G. Frank D. N. Mortin-Toth S. Robertson C. E. Feazel L. M. Rolle-Kampczyk U.. (2013). Sex differences in the gut microbiome drive hormone-dependent regulation of autoimmunity. Science 339, 1084–1088. 10.1126/science.1233521 23328391

[B25] Org E. Mehrabian M. Parks B. W. Shipkova P. Liu X. Drake T. A.. (2016). Sex differences and hormonal effects on gut microbiota composition in mice. Gut Microbes 7, 313–322. 10.1080/19490976.2016.1203502 27355107 PMC4988450

[B26] Plestilova L. Hrouzkova E. Burda H. Sumbera R. (2016). Does the morphology of the ear of the Chinese bamboo rat (Rhizomys sinensis) show “Subterranean” characteristics? J. Morphol. 277, 575–584. 10.1002/jmor.20519 26880690

[B27] Qu Q. Lu S. Li Z. Zhang J. Wang X. Zheng H.. (2023). The relationship between the preference of mating type (MAT) and source in the opportunistic pathogen Talaromyces marneffei. Med. Mycol. 61, myad027. 10.1093/mmy/myad027 36931899

[B28] Quast C. Pruesse E. Yilmaz P. Gerken J. Schweer T. Yarza P.. (2013). The SILVA ribosomal RNA gene database project: improved data processing and web-based tools. Nucleic. Acids Res. 41, D590–596. 10.1093/nar/gks1219 23193283 PMC3531112

[B29] Ridlon J. M. Ikegawa S. Alves J. M. Zhou B. Kobayashi A. Iida T.. (2013). Clostridium scindens: a human gut microbe with a high potential to convert glucocorticoids into androgens. J. Lipid Res. 54, 2437–2449. 10.1194/jlr.M038869 23772041 PMC3735941

[B30] Robinson M. D. McCarthy D. J. Smyth G. K. (2010). edgeR: a Bioconductor package for differential expression analysis of digital gene expression data. Bioinformatics 26, 139–140. 10.1093/bioinformatics/btp616 19910308 PMC2796818

[B31] Sanchez B. Hevia A. Gonzalez S. Margolles A. (2015). Interaction of intestinal microorganisms with the human host in the framework of autoimmune diseases. Front. Immunol. 6, 594. 10.3389/fimmu.2015.00594 26635808 PMC4653298

[B32] Sari W. N. Safika D. Fahrimal Y. (2017). Isolation and identification of a cellulolytic Enterobacter from rumen of Aceh cattle. Vet. World 10, 1515–1520. 10.14202/vetworld.2017.1515-1520 29391695 PMC5771179

[B33] Schnorr S. L. Candela M. Rampelli S. Centanni M. Consolandi C. Basaglia G.. (2014). Gut microbiome of the Hadza hunter-gatherers. Nat. Commun. 5, 3654. 10.1038/ncomms4654 24736369 PMC3996546

[B34] Wannaprasert T. (2018). Morphological characteristics of the tongue and lingual papillae of the large bamboo rat (*Rhizomys sumatrensis*). Anat. Sci. Int. 93, 323–331. 10.1007/s12565-017-0414-x 28952063

[B35] Xiao K. Liang X. Lu H. Li X. Zhang Z. Lu X.. (2022). Adaptation of gut microbiome and host metabolic systems to lignocellulosic degradation in bamboo rats. ISME J. 16, 1980–1992. 10.1038/s41396-022-01247-2 35568757 PMC9107070

[B36] Xiao L. Estelle J. Kiilerich P. Ramayo-Caldas Y. Xia Z. Feng Q.. (2016). A reference gene catalogue of the pig gut microbiome. Nat. Microbiol. 1, 16161. 10.1038/nmicrobiol.2016.161 27643971

[B37] Xie F. Jin W. Si H. Yuan Y. Tao Y. Liu J.. (2021). An integrated gene catalog and over 10,000 metagenome-assembled genomes from the gastrointestinal microbiome of ruminants. Microbiome 9, 137. 10.1186/s40168-021-01078-x 34118976 PMC8199421

[B38] Xie G. Wang X. Zhao A. Yan J. Chen W. Jiang R.. (2017). Sex-dependent effects on gut microbiota regulate hepatic carcinogenic outcomes. Sci. Rep. 7, 45232. 10.1038/srep45232 28345673 PMC5366919

[B39] Xu Y. Liu X. Tu F. (2016). Complete mitochondrial genome of Chinese bamboo rat, *Rhizomys sinensis* and species divergence comparison. Mitochondrial. DNA A DNA Mapp. Seq. Anal. 27, 1773–1774. 10.3109/19401736.2014.963806 25264842

[B40] Yang Y. Qu C. Liang S. Wang G. Han H. Chen N.. (2018). Estrogen inhibits the overgrowth of Escherichia coli in the rat intestine under simulated microgravity. Mol. Med. Rep. 17, 2313–2320. 10.3892/mmr.2017.8109 29207065 PMC5783461

[B41] Yurkovetskiy L. Burrows M. Khan A. A. Graham L. Volchkov P. Becker L.. (2013). Gender bias in autoimmunity is influenced by microbiota. Immunity 39, 400–412. 10.1016/j.immuni.2013.08.013 23973225 PMC3822899

[B42] Zhan X. Hu Y. Yin Y. Wei F. Bruford M. W. (2009). Microsatellite loci for the Chinese bamboo rat *Rhizomus sinensis*. Mol. Ecol. Resour. 9, 1270–1272. 10.1111/j.1755-0998.2009.02664.x 21564899

[B43] Zhang N. N. Jiang Z. M. Li S. Z. Yang X. Liu E. H. (2023). Evolving interplay between natural products and gut microbiota. Eur. J. Pharmacol. 949, 175557. 10.1016/j.ejphar.2023.175557 36716810

[B44] Zhang X. Liao Y. Qin T. Ma J. Liu J. Zou J.. (2022). Developmental stage variation in the gut microbiome of South China tigers. Front. Microbiol. 13, 962614. 10.3389/fmicb.2022.962614 36439793 PMC9682017

[B45] Zhang Z. F. Nan Z. B. (2012). First report of *Erwinia persicinus* causing wilting of medicago sativa sprouts in China. Plant Dis. 96, 454. 10.1094/PDIS-10-11-0909 30727103

[B46] Zhao J. Shao T. Chen S. Tao X. Li J. (2021). Characterization and identification of cellulase-producing *Enterococcus species* isolated from Tibetan yak (*Bos grunniens*) rumen and their application in various forage silages. J. Appl. Microbiol. 131, 1102–1112. 10.1111/jam.15014 33484057

[B47] Zhao W. Wang T. Ren G. Li J. Tan F. Li W.. (2023). Molecular detection of Enterocytozoon bieneusi in farmed Asiatic brush-tailed porcupines (*Atherurus macrourus*) and bamboo rats (*Rhizomys pruinosus*) from Hainan Province, China: common occurrence, wide genetic variation and high zoonotic potential. Acta Trop. 242, 106915. 10.1016/j.actatropica.2023.106915 36997011

